# Urinary manifestations in African American and Caucasian inflammatory bowel disease patients: a retrospective cohort study

**DOI:** 10.1186/s12894-021-00951-z

**Published:** 2022-01-04

**Authors:** Jake Herbert, Emily Teeter, Landen Shane Burstiner, Ralfi Doka, Amor Royer, Anna H. Owings, Julia Liu, Sarah C. Glover, Pegah Hosseini-Carroll

**Affiliations:** 1 Dr. Kiran C. Patel College of Osteopathic Medicine, Nova Southeastern University, Davie, FL USA; 2 College of Liberal Arts and Sciences, University of Florida, Gainesville, FL USA; 3 Department of Internal Medicine, University of Mississippi Medical Center, Jackson, MS USA; 4 Division of Gastroenterology, Morehouse School of Medicine, Atlanta, GA USA; 5 Department of Digestive Disease, University of Mississippi Medical Center, Jackson, MS USA

**Keywords:** Inflammatory bowel disease, Urinary tract infection, Cystitis, Urolithiasis

## Abstract

**Background:**

Inflammatory bowel diseases (IBD), like ulcerative colitis (UC) and Crohn’s disease (CD), are associated with urinary extra-intestinal manifestations, like urolithiasis and uncomplicated urinary tract infections (UTIs). The literature reviewed for this study identifies an increased association of CD and urolithiasis against the general population as well as UC. Furthermore, the rates in which urinary comorbidities manifest have not been well characterized in cross-race analyses. The purpose of this study is to establish the prevalence of common urinary extra-intestinal manifestations in CD and UC and to further determine at what rate these affect the African American and Caucasian populations.

**Methodology:**

This is a retrospective cohort study using de-identified data collected from a research data base that included 6 integrated facilities associated with one tertiary healthcare center from 2012 to 2019. The electronic chart records for 3104 Caucasian and African American IBD patients were reviewed for frequency of urolithiasis and uncomplicated UTI via diagnosed ICD-10 codes. Comparison between data groups was made using multivariate regressions, t-tests, and chi square tests.

**Results:**

Our study included 3104 patients of which 59% were female, 38% were African American, and 43% were diagnosed with UC. Similar proportions of UC and CD diagnosed patients developed urolithiasis (6.0% vs 6.7%, *p* = 0.46), as well as uncomplicated UTIs (15.6% vs. 14.9%, *p* = 0.56). Similar proportions of African American and Caucasian patients developed urolithiasis (5.4% vs 7.0%, *p* = 0.09), but a higher proportion of African Americans developed uncomplicated UTIs (19.4% vs 12.6%, *p* ≤ 0.001).

**Conclusion:**

We found similar rates of urolithiasis formation in both UC and CD in this study. Furthermore, these rates were not significantly different between African American and Caucasian IBD populations. This suggests that UC patients have an elevated risk of urolithiasis formation as those patients with CD. Additionally, African Americans with IBD have a higher frequency of uncomplicated UTI as compared to their Caucasian counterparts.

## Background

Inflammatory bowel diseases (IBD), like ulcerative colitis (UC) and Crohn’s disease (CD), are associated with numerous extra-intestinal manifestations. Commonly studied urinary extra-intestinal manifestations found in CD include urolithiasis, urinary tract infections (UTI), and cystitis. However, these rates have not been well characterized in cross-race analyses.

The literature reviewed for this study identifies an association between CD and urolithiasis, specifically calcium oxalate stones and urate stones. Some have shown that the incidence of urolithiasis in CD ranges from 4 to 23% and that there is a 10–100 times increased risk of urolithiasis in CD patients as compared to the general population and UC patients [1]. The mechanism behind the increased calcium oxalate stone formation in CD is related to an increase in urinary oxalate excretion that is directly related to bile salt malabsorption. Under normal conditions oxalate is bound to calcium in the intestinal lumen, which limits the amount of oxalate absorbed in the intestines. In patients with a diseased or resected ileum, bile salts are poorly reabsorbed in the intestines leading to steatorrhea. The unabsorbed fats in the steatorrhea bind to the free calcium in the lumen and allow the intestines to absorb the excess oxalate [2]. Urate stones have been shown to be related to both lengthy diarrheal illnesses and surgeries that create small bowel ostomies. It is believed that the reason urate stones form after small bowel ostomies is due to dehydration and metabolic acidosis associated with the procedure. These stones form from acidic urine that results from intestinal fluid and bicarbonate loss allowing for urate stones to precipitate even if the patient’s urate concentration is not elevated [2, 3].

UC is a chronic disease that is accompanied by intermittent diarrhea, which puts these patients at increased risk of urate renal calculi. Regardless of the disease severity, active UC represents a significant risk factor for the formation of renal calculi [4]. Studies have shown that asymptomatic nephrolithiasis in the UC and CD community is so common that IBD patients with renal dysfunction should be screened for kidney stones to prevent severe complications like UTIs, renal failure, and sepsis [4, 5].

Besides nephrolithiasis, uncomplicated UTIs, defined as cystitis and lower UTIs, are also associated with urinary extra-intestinal complications in IBD. Cystitis is the most common urinary problem in patients with CD. Furthermore, the most severe complications from cystitis occur in male patients, even though most of the affected patients are female [6, 7]. CD patients with perianal disease, which includes perianal abscesses and anal fistulas, have been shown to have an increased risk for UTIs by promoting bacterial translocation from the perineum to the bladder. In UC, independent predictors for UTI formation were disease duration over 11 months and age over 40 years old, possibly due to a degree of immunosuppression in elderly people. Even though IBD patients have an increased risk of UTIs, there were no significant differences between the rate of UTI diagnoses between CD and UC [8].

Medications used to treat IBD can potentially influence the frequency of urinary extra-intestinal manifestations. It has been well documented that steroids increase the risk of infections and that their use in preoperative IBD patients undergoing elective bowel surgery have been associated with increased risk of infections [9, 10]. Other commonly used medications used to treat IBD include mesalamine, thiopurines, and anti-TNF monoclonal antibodies. Mesalamine is primarily active on the intestinal mucosa where it is believed to exerts its anti-inflammatory effect via the peroxisome proliferator-activated receptor (PPAR)-y [11]. Even though mesalamine is considered a relatively safe drug, it can lead to further precipitation of renal stones [12]. Anti-TNF monoclonal antibodies work differently by blocking the inflammatory molecule called tumor necrosis factor. Two of the most common anti-TNF antibodies are infliximab and adalimumab and it has been shown that the fourth most common site of infection in this class of medication was the urinary tract [13, 14].

Racial disparities in urinary comorbidities have not been well characterized in cross-race analysis and, when they have been studied, there are conflicting results. For instance, one study has shown that urolithiasis disproportionately affects Caucasian patients and that there is a 1.7% reduced risk of nephrolithiasis in African Americans [15]. Another study has shown that, after the age of 25, kidney stones become more common among men and that the incidence in African Americans increased 12% more than Caucasians every five years, regardless of gender [16]. When it comes to UTI incidence and prevalence among African Americans there has been little data examining the general population and most of the literature tends to examine the incidence in children, febrile infants, and pregnant mothers.

There are two purposes of this study and the first one is to establish the frequency of common urinary extra-intestinal manifestation in CD and UC. The second purpose is to determine at what rate these urinary extra-intestinal manifestations affect the African American and Caucasian IBD populations.

## Methodology

### Database and study population

This study was approved under University of Mississippi Medical Center (UMMC) IRB #2019-0194 for retrospective analysis of 3272 identified charts of individuals with either UC or CD. This study was designed using a retrospective cohort model. The data was collected by IRB-approved researchers from 2012 to 2019 from a research data base that recorded data from 6 integrated facilities associated with one tertiary healthcare center based around Jackson, Mississippi. The study data was collected from EPIC, de-identified, and managed using Research Electronic Data Capture (REDCap) tools hosted at UMMC [17, 18]**.**

### Study design

The de-identified electronic chart records for 3104 Caucasian and African American patients that were diagnosed with CD or UC were reviewed, as determined by the ICD-10 codes K50 or K51, respectively. After determining whether the patients were diagnosed with CD or UC, the patients’ charts were further reviewed for any occurrence of urolithiasis, specifically kidney stones and ureter stones via ICD-10 codes N20. Patients with the ICD-10 codes N21, which represented bladder stones, were not included in the urolithiasis group in this study. The patient charts were then further reviewed for any occurrence of uncomplicated urinary tract infections as determined by the ICD-10 codes N30.0 and N39.0, which represent cystitis and unspecified UTI, respectively.

The patients’ socio-demographics, which included their age, gender, zip codes, and race, were extracted from their charts by IRB-approved researchers. The study included 3272 patients, but 168 patients were excluded due to incomplete demographic data, registration and coding errors, or incorrect diagnosis of IBD. Additional information about prior gastrointestinal surgeries, the number of corticosteroids and aminosalicylates prescribed, and the presence of fistulizing disease phenotype were also extracted from the patients’ charts. Due to the inconsistency in the number of stones collected for pathology analysis as well as the number of urinary samples sent for culture, the type of stones and UTI bacteria were not examined in this study. Afterwards, the IRB-approved researchers randomly selected 100 charts to manually reviewed to verify the accuracy of the data.

### Statistical analysis

Continuous variables were evaluated using Student’s t-test. Categorical variables were evaluated using Chi-square and Fisher exact test. Univariate logistic regression models were fit to examine associations between covariates of interest and the occurrence of urolithiasis. Covariates with *p* < 0.20 in the univariate analyses were included in a multivariate model. Patients with an occurrence of cystitis or an unspecified UTI were grouped together into the category “uncomplicated UTI.” The above process was repeated to evaluate the relationship between covariates of interest and the occurrence of uncomplicated UTIs. Statistical analyses were performed using STATA v16 (College Station, TX).

## Results

Our study included 3104 patients of which 38% were African American, 62% were Caucasian, 59% were female, 41% were male, 57% were diagnosed with CD, and 43% were diagnosed with UC. The average age of the patient population was 46.7 years and the average income of the 3049 patients with a recorded zip code was $47,165. There was 1152 African Americans and 1897 Caucasian with a recorded zip code. The average of the estimated median household income for the African American population was $40,443 and the average of the estimated median household income for the Caucasian population was $51,247 (*p* = 0.001). This study examined the average number of corticosteroids prescribed per patient, which was 1.56, along with the proportion of patients that were prescribed either corticosteroids or aminosalicyclates, which was 34% and 32%, respectively.

**Fig. 1:**
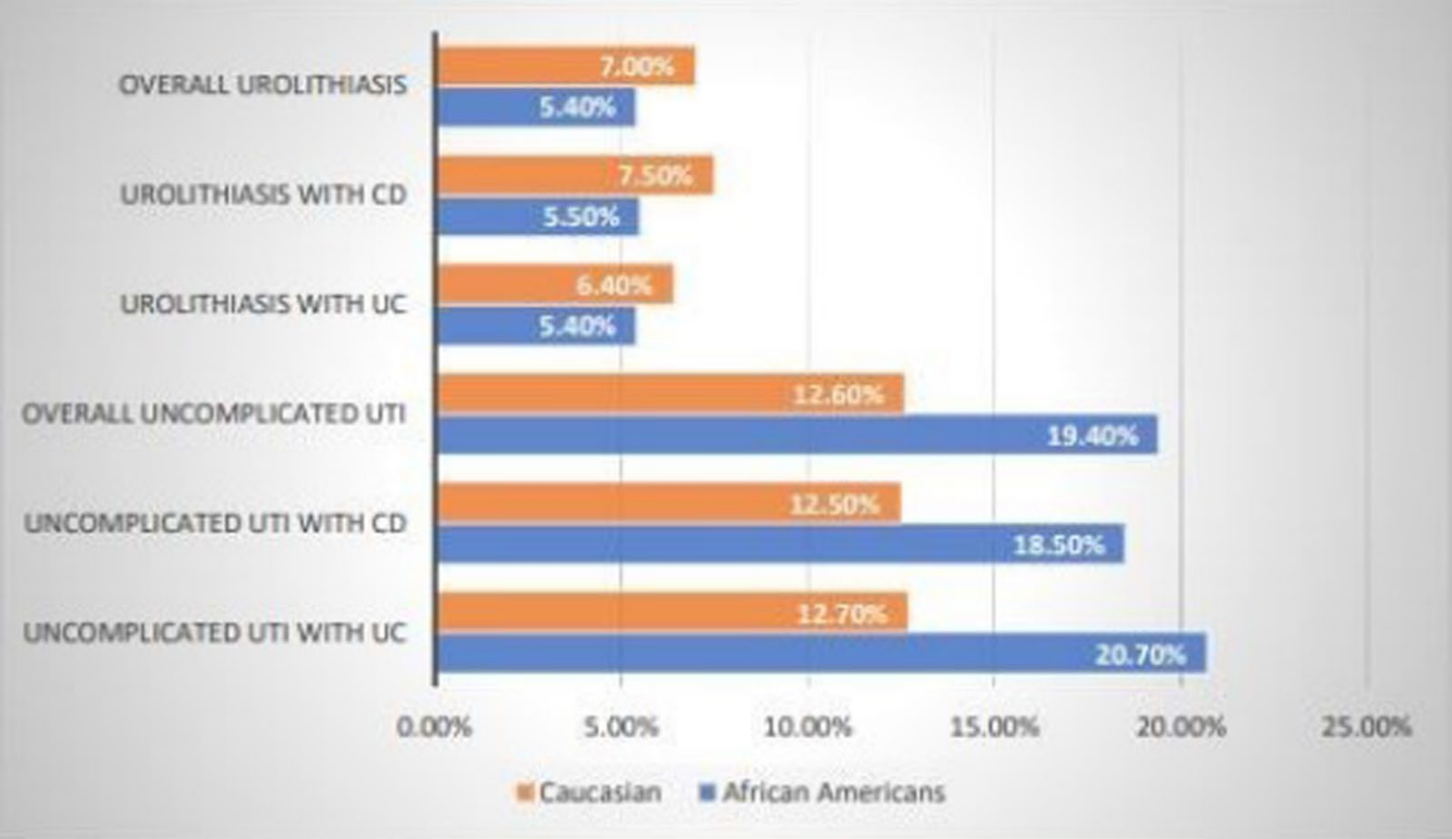
Urinary complications in African Americain and Caucasian IBD patients

Table 1 and Fig. 1 summarizes the findings between race and urinary complications. The prevalence of urolithiasis was comparable among African American and Caucasian patients (5.4% vs 7.0%, *p* = 0.09) with African Americans showing a higher risk of developing uncomplicated UTIs (19.4% vs 12.6%, *p* ≤ 0.001). Additionally, there were similar proportions of African Americans and Caucasians with CD who developed urolithiasis (5.5% vs 7.5%, *p* = 0.11). However, African Americans with CD had a higher risk of developing uncomplicated UTIs (18.5% vs. 12.5%, *p* = 0.001). The prevalence of urolithiasis was comparable among African Americans with UC and Caucasians with UC (5.4% vs. 6.4%, *p* = 0.44), while African Americans with UC showed an elevated risk of developing uncomplicated UTIs (20.7% vs. 12.7%, *p* ≤ 0.001).

**Table 1:** Baseline characteristics between race and urinary manifestations

	African Americans N = 1175	Caucasian Americans N = 1929	*p* value	Total N = 3104
Mean age ± SD	47.7 ± 19.2	46.2 ± 19.9	0.042	46.7 ± 19.7
Female sex, N (%)	732 (62%)	1110 (58%)	0.009	1842 (59%)
Crohn’s disease	691 (59%)	1087 (56%)	0.18	1778 (57%)
Ulcerative colitis	484 (41%)	842 (44%)	0.18	1326 (43%)
Average # of corticosteroid prescriptions ± SD	1.8 ± 4.4	1.41 ± 3.6	0.008	1.56 ± 3.9
# of patients prescribed Aminosalicylates, N (%)	348 (30.2%)	638 (33.6%)	0.050	986 (32.3%)
# of patients prescribed corticosteroid. N (%)	413 (35.9%)	633 (33.4%)	0.16	1046 (34.3%)
Overall urolithiasis, N (%)	64 (5.4%)	135 (7.0%)	0.09	199 (6.4%)
Overall uncomplicated urinary tract infections, N (%)	228 (19.4%)	243 (12.6%)	< 0.001	471 (15.2%)
Urolithiasis with CD, N (%)	38 (5.5%)	81 (7.5%)	0.11	119 (6.7%)
Uncomplicated urinary tract infections with CD, N (%)	128 (18.5%)	136 (12.5%)	0.001	264 (14.9%)
Urolithiasis with UC, N (%)	26 (5.4%)	54 (6.4%)	0.44	80 (6.0%)
Uncomplicated urinary tract infections with UC, N (%)	100 (20.7%)	107 (12.7%)	< 0.001	207 (8.4%)

**Fig. 2:**
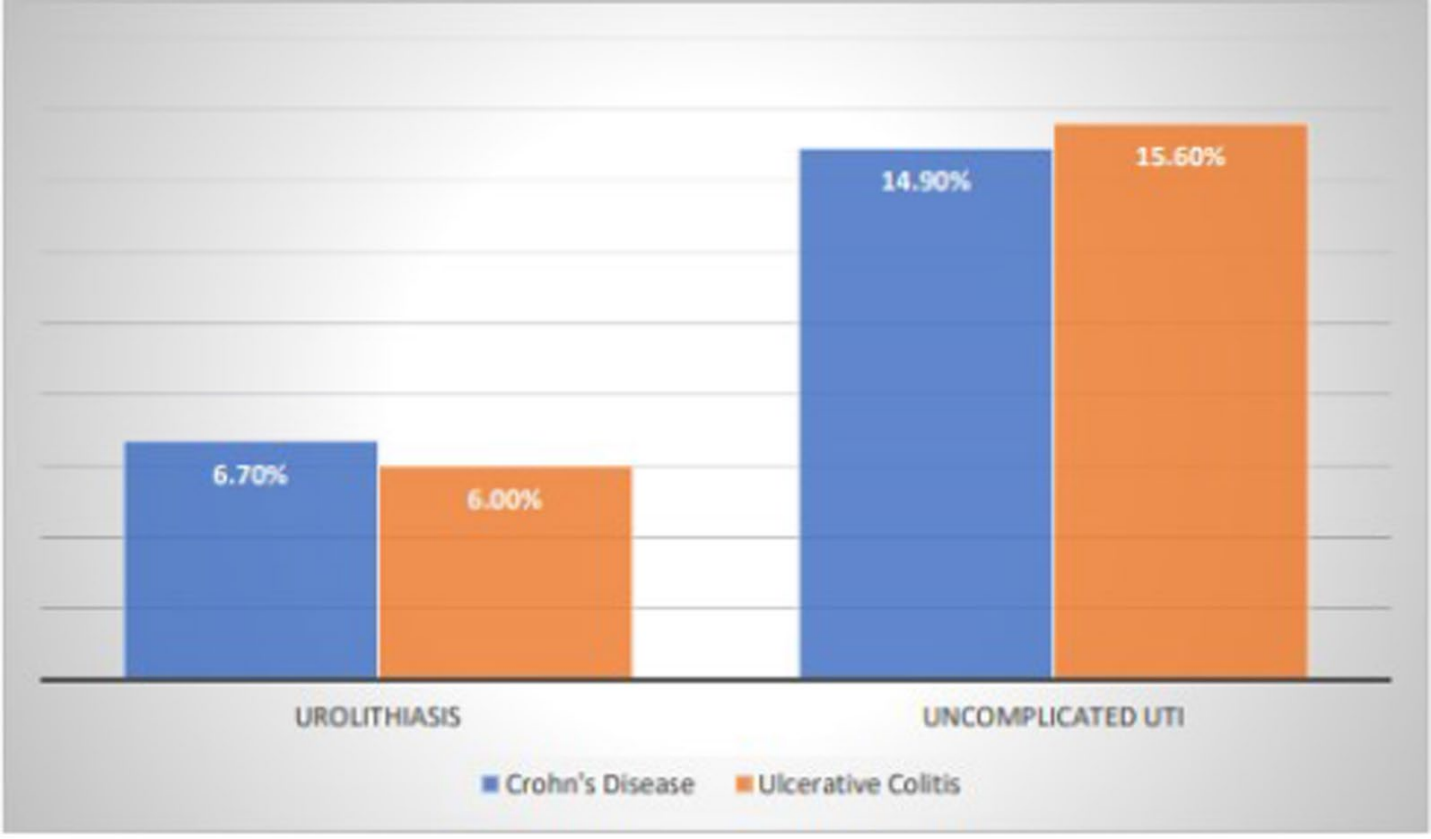
Urinary complications in Crohn’s disease and ulcerative colitis

Table 2 and Fig. 2 summarizes the findings between the IBD subtypes and urinary complications. Our findings showed there were similar proportions of UC and CD patients who developed urolithiasis (6.0% vs 6.7%, *p* = 0.46), as well as uncomplicated UTIs (15.6% vs 14.9%, *p* = 0.56). The proportion of patients prescribed aminosalicylates was higher in UC than CD (38% vs 27%, *p* ≤ 0.001). However, the proportion of patients prescribed corticosteroids was higher in CD than UC (36% vs 31%, *p* = 0.003).

**Table 2:** IBD characteristics and urinary manifestations

	Crohn’s disease N = 1778	Ulcerative colitis N = 1326	*p* value	Total N = 3104
Mean age ± SD (years)	46.6 ± 19.7	46.9 ± 19.7	0.61	46.7 ± 19.7
Female sex, N (%)	1055 (59%)	787 (59%)	0.99	1842 (59%)
African American, N (%)	691 (39%)	484 (37%)	0.18	1175 (38%)
Average # of corticosteroid prescriptions ± SD	1.5 ± 3.6	1.58 ± 4.3	0.70	1.56 ± 3.9
# of patients prescribed aminosalicylates, N (%)	478 (27%)	508 (38%)	< 0.001	986 (32%)
# of patients prescribed corticosteroid, N (%)	639 (36%)	407 (31%)	0.003	1046 (34%)
Urolithiasis, N (%)	119 (6.7%)	80 (6.0%)	0.46	199 (6.4%)
Uncomplicated urinary tract infections, N (%)	264 (14.9%)	207 (15.6%)	0.56	471 (15.2%)

As seen in Table 3, after adjusting for covariates, history of GI surgery (OR 2.495 [1.598–3.897], *p* < 0.001), history of prednisone prescription (OR 1.729 [1.282–2.333], *p* < 0.001), and caucasian race (OR 1.492 [1.087–2.048], *p* = 0.013) were associated with increased odds of urolithiasis. The analysis exhibited that sex, age, type of IBD, and history of aminosalicylate prescription were not significantly associated with urolithiasis.

**Table 3:** Odds ratios of covariates on diagnosis of urolithiasis and uncomplicated UTI

Urolithiasis—univariate model	Odds ratio	P > Z	95% confidence interval
Caucasian race	1.306	0.088	0.961–1.775
Female sex	1.142	0.378	0.850–1.536
IBD subtype: ulcerative colitis	0.895	0.458	0.668–1.200
History of GI surgery	2.706	< 0.001	1.749–4.187
History of aminosalicylates prescription	0.879	0.424	0.640–1.206
History of prednisone prescription	1.834	< 0.001	1.367–2.462
Age	1.005	0.195	0.998–1.012

Urolithiasis—multivariate model	Odds ratio	P > Z	95% confidence interval
History of GI surgery	2.495	< 0.001	1.598–3.897
History of prednisone prescription	1.729	< 0.001	1.282–2.333
Caucasian race	1.492	0.013	1.087–2.048
Age	1.005	0.233	0.997–1.012

Uncomplicated UTI– univariate model	Odds ratio	P > Z	95% confidence interval
Age	1.004	0.14	0.999–1.009
Caucasian race	0.599	< 0.001	0.491–0.729
Female sex	3.791	< 0.001	2.955–4.863
History of GI surgery	3.957	< 0.001	2.893–5.412
IBD Subtype: Ulcerative Colitis	1.061	0.56	0.871–1.293
History of aminosalicylate prescription	0.980	0.85	0.793–1.211
History of prednisone prescription	1.790	< 0.001	1.460–2.194
History of fistulizing disease (only Crohn’s disease patients)	2.957	< 0.001	2.145 -4.076

Uncomplicated UTI—multivariate model	Odds ratio	P > Z	95% confidence interval
Age	1.003	0.26	0.998–1.008
Caucasian race	0.665	< 0.001	0.540–0.819
Female sex	4.135	< 0.001	3.187–5.365
History of GI surgery	3.876	< 0.001	2.769–5.426
History of prednisone prescription	1.667	< 0.001	1.345–2.066
History of fistulizing disease (only CD patients)	2.003	< 0.001	1.366–2.939

After adusting for covariates, female sex (OR 4.135 [3.187–5.365], *p* < 0.001), history of GI surgery (OR 3.876 [2.769–5.426], *p* < 0.001), and history of prednisone prescription (OR 1.667 [1.345–2.066], *p* < 0.001) were associated with increased odds of uncomplicated UTI. Additionally, fistula formation among CD patients was associated with increased odds of uncomplicated UTI (OR 2.003 [1.366–2.939], *p* < 0.001). In contrast to urolithiasis, caucasian race was associated with decreased odds of uncomplicated UTI (OR 0.665 [0.540–0.819], *p* < 0.001). Age, type of IBD, and history of aminosalicylate prescription were not significantly associated with uncomplicated UTI.

## Discussions

In this retrospective cohort study of urinary extra-intestinal complications between CD and UC in the African American and Caucasian populations, we found similar prevalence of urolithiasis in UC and CD patients. Nephrolithiasis has been considered a hallmark symptom of CD, while only, a small number of studies have shown an increased prevalence in kidney stone formation among UC patients against the general population [1, 19]. While our CD population had slightly more kidney stones than the UC population, the difference was marginal. We also found increased frequency of uncomplicated UTI in African American IBD patients compared to Caucasian IBD patients, while the rate of uncomplicated UTI in CD and UC were similar.

Our data suggests additional studies are needed in UC patients. The pathology behind kidney stone formation in CD has been elucidated, but a specific pathology in UC remains unclear. It is plausible that, because UC is associated with persistent diarrhea, urate stones may form more commonly in this population due to dehydration and bicarbonate loss. UC patients who have undergone surgical treatment are also at risk of developing urolithiasis due to alterations in sodium excretion as surgery lowers the urinary output and increases renal tubule reabsorption of sodium [20].

Another possible explanation may involve the difference in medications. 1%-2% of all renal stones are drug-induced and they are frequently underdiagnosed. Drugs that have been associated with stone formation include diuretics, protease inhibitors, anti-convulsants, certain antibiotics, and mesalamine, which is a commonly used anti-inflammatory drug prescribed to UC patients and those with mild cases of CD. The mechanism of mesalamine-induced urolithiasis formation is poorly understood but it is thought to be induced by high urine excretion of the mesalamine along with its poor urinary saturation which are secondary metabolic drug effects. Crystallized mesalamine stones are soft, orange-brown stones that are radiolucent on computer tomography imaging and often show no dilation of the ureters, making them difficult to diagnose [12]. As seen in Table 2, patients with UC received significantly more aminosalicylate prescriptions than those with CD. However, we also found that aminosalicylate prescription was not associated with an increased odds of urolithiasis formation.

CD patients may experience fistulizing disease, and as our study shows fistulas are associated with a 2.003 increased risk of uncomplicated UTI, so one may expect CD patients to have a significantly higher frequency of UTI than UC patients. However, this was not the case in our study. Despite the lack of fistulas in the UC population, they had a similar rate of UTIs. This could partially be explained by the mean age of the UC patients, which was 46.9. Peyrin-Bioroulet [8] has found that one major independent risk factor for UTI development in UC patients is being over the age of 40 since this could mark the decline of immunocompetency in these patients. Other risk factors that could explain the increased rate of UTIs in both the UC population and the CD population include the high prevalence of anorectal complications, which has been shown to increase the risk for UTIs by promoting bacterial translocation from the perineum to the bladder [8]. For patients with CD the most common anorectal complications are fistula and abscess formation, and for patients with UC the most common anorectal complication is abscess formation, with studies showing up to 5% of chronic UC patients having this complication [21]. Patients with anorectal complications also require earlier and more aggressive medication management, including steroids and biological therapies, both of which may be correlated with higher rates of infections [10, 13, 14, 21].

The increased rate of UTIs in African Americans could be tied to health care disparities that affect this population. We used zip codes to estimate the median household income of our specific cohort, and, on average, African Americans made about $11,000 less annually. Compared to Caucasians, African Americans are less likely to be able to afford private health insurance and are twice as likely to be uninsured. African Americans are more likely to work in jobs that do not provide health insurance and they are less likely to have a consistent source of healthcare [22]. With the lack of a primary healthcare source and a lower median income, African Americans are twice as likely to present to the emergency department with UTI symptoms, which could explain why African Americans in our population had a higher rate of uncomplicated UTIs [23]. Another explanation for the increased rate of UTIs in African Americans could be related to their diet. African Americans have diets high in salt and fat which makes it easier for African Americans to develop a high body mass index [24]. A high body mass index has been shown to be associated with an increased risk of UTI formation, as well as more severe infections like pyelonephritis, particularly when the body mass index is greater than 30 [25]. Further information of the patients’ body mass index at the time of UTI confirmation could help clarify these findings, as well as a study designed to examine the differences between Caucasian and African American diets.

Due to our large cohort, which is spread out among many physicians and several medical centers, our analysis is limited by the use of ICD-10 codes for the diagnosis of uncomplicated UTIs and urolithiasis. While 100 patient charts were manually reviewed by IRB-approved researchers to confirm accuracy of the coding, it would have been nearly impossible to confirm all of the diagnoses, and this may present another limitation of our study. We did not personally review the computer tomography scans that reported the kidney stones, as well as the data of the urinalysis cultures that confirmed the UTIs in CD and UC patients. We also did not examine the types of stones that were formed in patients with CD versus the types of stones that formed in patients with UC. Our study focused on the number of patients who have ever been diagnosed with an UTI. It would be interesting to further analyze this data to see the average number of UTIs per person. Further studies should be conducted to examine the rate at which different types of kidney stones are formed in UC and the rates at which different pathogens are cultured in IBD patients with UTIs. It would also be interesting to see how the rates of urolithiasis and UTIs in this IBD population compare to the general population with similar comorbidities. Additional studies should also examine whether IBD patients with urolithiasis have an elevated risk of developing further kidney complications, such as pyelonephritis and renal failure. It may also be helpful to have further studies stratify the IBD population based on the severity of their disease.

## Conclusion

This study found that there were similar rates of urolithiasis formation in both UC and CD. Furthermore, these rates were not significantly different between African American and Caucasian IBD populations. This could potentially mean that UC patients have a similar elevated risk of urolithiasis formation as those patients with CD. Additionally, there were similar rates of uncomplicated UTI formation in both UC and CD. However, African Americans with IBD have a significantly higher burden of uncomplicated UTI complications as compared to their Caucasian counterparts.

## Data Availability

The dataset generated and/or analyzed during the current study are not publicly available due to identifiable patient information but are available from the corresponding author upon reasonable request.

## Abbreviations

IBD: Inflammatory bowel disease
CD: Crohn’s disease
UC: Ulcerative colitis
UTI: Urinary tract infection
REDCap: Research Electronic Data Capture
